# Asthma exacerbations in a subtropical area and the role of respiratory viruses: a cross-sectional study

**DOI:** 10.1186/s12890-018-0669-6

**Published:** 2018-07-03

**Authors:** Lusmaia Damaceno Camargo Costa, Paulo Augusto Moreira Camargos, Paul L. P. Brand, Fabíola Souza Fiaccadori, Menira Borges de Lima Dias e Souza, Divina das Dôres de Paula Cardoso, Ítalo de Araújo Castro, Ruth Minamisava, Paulo Sérgio Sucasas da Costa

**Affiliations:** 1 Pediatric Pulmonology Unit, University Hospital, Federal University of Goiás, Primeira Avenida, S/N. Setor Leste Universitária, Goiânia, CEP: 746050-20 Brazil; 2 Pediatric Pulmonology Unit, University Hospital, Federal University of Minas Gerais, Belo Horizonte, Brazil; 3 Princess Amalia Children’s Centre, Isala Hospital, Zwolle, and UMCG Postgraduate School of Medicine, University Medical Centre and University of Groningen, Groningen, the Netherlands; 4 Human Virology Department, Public Health and Tropical Pathology Institute, Federal University of Goiás, Goiânia, Brazil; 5 Faculty of Nursing, Universidade Federal de Goiás, Goiânia, GO Brazil

**Keywords:** Asthma, Exacerbations, Virus, Child

## Abstract

**Background:**

Multiple factors are involved in asthma exacerbations, including environmental exposure and viral infections. We aimed to assess the association between severe asthma exacerbations, acute respiratory viral infections and other potential risk factors.

**Methods:**

Asthmatic children aged 4–14 years were enrolled for a period of 12 months and divided into two groups: those with exacerbated asthma (group 1) and non-exacerbated asthma (group 2). Clinical data were obtained and nasopharyngeal samples were collected through nasopharyngeal aspirate or swab and analysed via indirect fluorescent immunoassays to detect influenza A and B viruses, parainfluenza 1–3, adenovirus and respiratory syncytial virus. Rhinovirus was detected via molecular assays. Potential risk factors for asthma exacerbation were identified in univariate and multivariate analyses.

**Results:**

In 153 children (group 1: 92; group 2: 61), median age 7 and 8 years, respectively, the rate of virus detection was 87.7%. There was no difference between groups regarding the frequency of virus detection (*p* = 0.68); however, group 1 showed a lower frequency (19.2%) of inhaled corticosteroid use (91.4%, *p* < 0.01) and evidence of inadequate disease control. In the multivariate analysis, the occurrence of three or more visits to the emergency room in the past 12 months (IRR = 1.40; *p* = 0.04) and nonadherence to inhaled corticosteroid (IRR = 4.87; p < 0.01) were the only factors associated with exacerbation.

**Conclusion:**

Our results suggest an association between asthma exacerbations, poor disease control and nonadherence to asthma medication, suggesting that viruses may not be the only culprits for asthma exacerbations in this population.

## Background

Asthma exacerbations generate considerable morbidity, affecting various aspects of the patient’s quality of life, and producing high costs for the health system [1]. The multifactorial origin of asthma exacerbations has been well described and includes allergen exposure, acute viral respiratory tract infections, pollutants, climate changes, exercise, amongst other factors [1–3]. Despite the existence of effective drugs, poor asthma control remains common in many children worldwide [4]. A key characteristic of poor asthma control is the occurrence of asthma exacerbations, and a history of recent emergency room visits for asthma is a strong and independent predictor of future asthma exacerbations [5]. Asthma tends to be poorly controlled in the socially disadvantaged population [6] and the reasons are multifactorial with complex interactions [7]. On the other hand, authors have identified specific genetic variants associated with susceptibility to viral respiratory infections, severity of infection and virus-induced exacerbations of asthma during childhood [8].

Since the early 1970s, viral respiratory infections have been associated with the onset of asthma exacerbations. However, studies in children, who are particularly susceptible to viral infection due to their relative immunological immaturity, increased in the 1990s, when molecular techniques became more sensitive allowing for the identification of more respiratory viruses and facilitating a better understanding of this association [9–16]. Studies using *reverse transcription*-*polymerase chain reaction* (RT-PCR) detected respiratory viruses in more than 90% of asthma exacerbations [9].

Acute respiratory tract infections are responsible for high morbidity and mortality, accounting for around 20% of the estimated 9 million deaths of children worldwide in 2007, according to the World Health Organization. Viruses are responsible for most of these infections, causing generally mild and self-limited infections, though some may become very severe or complicate the clinical course of patients with underlying chronic lung diseases, including asthma [17]. Moreover, the association between viral infection and environmental exposure is described as a trigger for exacerbations and type 2 inflammation is associated with an increased risk of virus-induced exacerbations [18, 19]. Achieving asthma control remains a global challenge, especially in poor-resource populations. Although viral infection is an important trigger, few studies in this area have been conducted in tropical countries. To add more details about this possible association in Latin American children, we examined the viral detection rate in a group of Brazilian asthmatic children with and without exacerbation. We hypothesize that in a population of a low income country, living in a subtropical area, viruses may not be the main trigger of an asthmatic exacerbation.

## Methods

### Setting

This study was carried out in the city of Goiânia, capital of Goiás state, located in Central Brazil, a region with semi-humid tropical climate with an estimated population of 1,302,001 inhabitants. Public health care is provided free of charge by the Brazilian Unified Health System, and an estimated 70% of the population use the public health system [20].

### Study population

From June 2012 through August 2013, asthmatic children were screened for respiratory viruses if they met the following inclusion criteria: 4–14 year olds, admitted to emergency rooms of three hospitals, which attend children from public and private health insurance due to asthma exacerbation (group 1). We only included in the study patients who had at least three previous episodes of bronchospasm and fulfill the GINA criteria for asthma definition [1]. We did not include children under 4 years old because of the difficulty in differentiation of asthma from wheezing episodes of other origin in very young children. Exacerbations were defined as increased symptoms that required a change in medication as judged by the attending physician according to ATS/ERS statement [21]. Exacerbations requiring a course of oral corticosteroids or admission to hospital were considered severe.

Asthmatic children without exacerbation (group 2), were invited to participate in this study during their appointment in the major respiratory outpatient clinic of the city, which attend children from public health insurance (Brazilian Unified Health System). They were only eligible for inclusion when they reported not having symptoms of upper respiratory tract infection, such as rhinorrhoea, fever and nasal congestion in the 4 weeks prior to the clinic visit. All patients had similar access to primary care and maintenance medications. Exclusion criteria for both groups were premature birth and the presence of cardiorespiratory chronic diseases, neurologic and metabolic disorders and immunosuppression.

The study protocol was approved by the Clinical Research Ethics Committee (HC/UFG Protocol 175/2011). Written informed consent was obtained from parents, and written assent was obtained from older children (over 8 years old).

### Data and sample collection

For each child, data on sociodemographic data, asthma related symptoms, hospitalisations in the last 12 months and environmental exposure to aeroallergens and second-hand tobacco was collected by two researchers (LDCC and PSSC). A nasopharyngeal aspirate was obtained from each patient upon admission to the emergency ward (Group 1) or during the interview (Group 2). The nasopharyngeal swab specimens were obtained from the nostril from a depth of 2 to 3 cm by using a sterile ray swab that was then inserted into a vial containing 2.5 ml of viral transport medium (MEM). For the nasopharyngeal aspirate, a disposable catheter connected to a mucus extractor was inserted into the nostril to a depth of 5 to 7 cm and drawn back while applying gentle suction with an electric suction device [22]. Nasopharyngeal flocked swab (Chemicon-Millipore Corporation, Billerica, MA, USA) was obtained in cases of absence of sufficient sample by nasopharyngeal aspirate.

Samples were transported at 4 °C to the Human Virology Laboratory, Federal University of Goias, Tropical Medicine and Public Health Institute and processed immediately. The supernatant was stored at − 70 °C for molecular study, and the pellet was used in the immunofluorescence assay.

### Viral detection

Each sample was analysed using a Respiratory Panel I Viral Screening and Identification IFA Reagent immunofluorescence kit (Chemicon-Millipore Corporation, Billerica, MA, USA) consisting of a panel of monoclonal antibodies specific to influenza virus A (FLUVA), influenza virus B (FLUVB), human respiratory syncytial virus (hRSV), human adenovirus (hADV), and human parainfluenza viruses (hPIV) 1, 2, and 3 following the manufacturer’s instructions.

For molecular identification of rhinovirus, all samples were submitted to ribonucleic acid (RNA) extraction using a QIAamp® cador® Pathogen Mini Kit (Qiagen, Germany), and conventional RT-PCR was conducted using primers described previously [23]. Briefly, 20 μL of viral RNA was extracted using random hexamers (Random Primer – *Invitrogen*® life technologies, USA) in a final volume of 50 μL. The reaction was incubated at 25 °C for 5 min, 42 °C for 10 min, 50 °C for 20 min, and 85 °C for 5 min, all in a single cycle. For the polymerase chain reaction (PCR) reaction, the commercial kit Platinum PCR SuperMix HF (*Invitrogen*® life technologies, USA), 0.4 mM of each primer (P1–1 e P3–1), and 2.5 μL of cDNA were mixed and submitted to amplification in a thermocycler (Mastercycler Personal/Eppendorf) with the following cycling parameters: 94 °C for 2 min, followed by 35 cycles of 94 °C for 20 s, 53 °C for 30 s, 72 °C for 40 s, and a final extension of 72 °C for 10 min. The products were submitted to 1.5% agarose gel electrophoresis and visualised using a UV transilluminator. The samples were compared to a molecular marker (100 bp ladder), and the expected amplicon size for Rhinovirus was 390 bp. In all reactions, positive (previously sequenced Rhinovirus samples) and negative (MilliQ water) controls were used.

### Statistical analysis

Kruskal-Wallis, Chi-square and Fisher’s exact tests were used to compare medians and proportions between groups. Poisson multivariate regression analysis was performed to identify the variables associated with the outcome (asthma exacerbation), including the independent variables significantly or near-significantly (*p* ≤ 0.1) in bivariate analysis. Results are presented as the incidence risk ratio (IRR) with the 95% confidence interval (95% CI). All analyses were performed using STATA v 12.0 (Stata Corp, College Station, TX, USA).

## Results

During the study period, respiratory secretion samples were collected from 158 children. Five patients (3.2%) were excluded from analysis (three from group 1 and two from group 2) due to an insufficient amount of material for the examinations. Of the 153 samples analysed, 92 (60.1%) belonged to group 1 and 61 (39.9%) to group 2. The median age was 7.0 years (IQR =5.0–8.7) in group 1 and 8.0 years (6.0–11.0) in group 2. Table 1 shows clinical and socio-demographic characteristics of the patients in the study.

**Table 1:** Clinical and social demographic features of patients with and without exacerbation

	G1 (92)	G2 (61)	
	N (%)	N (%)	P
Male gender	56 (60.9)	42 (68.9)	0.20
White ethnicity	62 (67.4)	28 (45.9)	< 0.01
Monthly family income ≤200 US$	41 (44.6)	39 (63.9)	0.01
Maternal schooling level ≤ 8 years	47 (51.1)	25 (41.0)	0.14
≥ 3 emergency room visits in the past 12 months	73 (76.0)	23 (24.0)	< 0.01
1 or more hospitalisations for asthma in the past 12 months	58 (80.6)	14 (19.4)	< 0.01
Cough or dyspnoea on exertion (yes)	66 (75.0)	22 (25.0)	< 0.01
Nocturnal cough, in-between exacerbations (yes)	70 (80.5)	17 (19.5)	< 0.01
Use of ICS (yes)	6 (19.2)	84 (91.4)	< 0.01
Exposure to furry animals (yes)	58 (65.9)	30 (34.1)	0.09
Exposure to mould (yes)	44 (71.0)	18 (29.0)	0.02
Exposure to house dust mite (yes)	66 (59.5)	45 (40.5)	0.80
Exposure to second hand tobacco smoking (yes)	33 (56.9)	25 (43.1)	0.61

*G1* group 1(exacerbated asthmatics), *G2* group 2 (non-exacerbated asthmatics), *ICS* inhaled corticosteroids

In 134 children (87.6%), the nasal sample was obtained by nasopharyngeal aspirate and in 19 children (12.4%) by nasal swab. Most patients (72.0%) were recruited between April and June (autumn in the southern hemisphere); the highest detection rate of viruses (63.2%) also occurs during this time period.

Of the 153 samples tested, 136 were positive (88.9%; 95% CI 83.1–93.2) for at least one virus, and the detection rate of viruses was similar in both groups (*p* = 0.7). More than one viral agent was identified in 27.9% (36) of the samples, with no difference between groups (*p* = 0.1). The most common virus was hRV (82.4, 95% CI 75.77–87.8), followed by FLUVA (15.0%), hADV (6.5%), hPIV2 (4.6%), hRSV (3.9%), FLUVB (2.6%) and hPIV1 (2.6%). None of the samples were positive for hPIV3. FLUVB and hPIV1 were only found with other viruses (Table 2).

**Table 2:** Rates of virus detection and co-detection between groups

Virus	G1	G2	Total	
	N (%)	N (%)	N (%)	*P*
hRV	74 (80.4)	52 (85.2)	126 (82.4)	0.19
FLUVA	17 (18.5)	6 (9.8)	23 (15.0)	0.17
hAdV	5 (5.4)	5 (8.2)	10 (6.5)	0.30
HPIV2	6 (6.5)	1 (1.6)	7 (4.6)	0.20
hRSV	5 (5.4)	1 (1.6)	6 (3.9)	0.30
FLUV B	3 (3.3)	1 (1.6)	4 (2.6)	0.50
HPIV1	4 (4.3)	0 (0.0)	4 (2.6)	0.16
Any detected virus	81 (88.0)	55 (90.2)	136 (88.9)	0.70
Co-detection	27 (20.9)	9 (7.0)	36 (27.9)	0.10

*G1* group 1, *G2* group 2, *hRV* human rhinovirus, *FLUVA* influenza virus A, *hAdV* human adenovirus, *FLUVB* influenza virus B, *hRSV* human respiratory syncytial virus, *HPIV2* human parainfluenza virus type 2, *HPIV1* parainfluenza virus type 1

We performed an analysis of the severity of exacerbations and did not find statistically significant differences between the groups with and without virus infection regarding severity, such as hospitalization (*p* = 0.4), oxygen use (*p* = 0.8), and systemic use of corticosteroids (*p* = 0.5).

There were no differences in the viral rates between children with and without exacerbation. The results of univariate analysis evidenced that some variables were associated with asthma exacerbation: at least one hospitalisation for asthma in the past 12 months, at least three emergency room visits in the past 12 months, white ethnicity, monthly family income below 200 US$, cough or dyspnoea on exertion, nocturnal cough, exposure to mould and poor medication adherence (Table 1). After multivariate analysis, at least three visits to hospital for asthma in the last 12 months and nonadherence in inhaled corticosteroids (ICS) remained associated with the outcome (Table 3).

**Table 3:** Variables associated with asthma exacerbation, multivariate analysis final model

Variables	IRR	95% CI	*p*-value
≥ 3 visits for asthma, 12 months	1·40	1·01–1·95	0·04
Nonadherence to inhaled corticosteroids	4·87	2·43–9·76	< 0·01

## Discussion

In this study, we assessed asthmatic children in emergency room and in an outpatient clinic from a tropical region in Brazil and the results were consistent with those observed by several authors around the world, with high viral detection rates [9–11], with rhinovirus being the most frequent agent similar with others studies [9, 12, 15, 16, 23–25]. However the presence of respiratory viruses was similar in children with and without asthma exacerbation (regardless of severity) and although most studies have shown a higher prevalence of viruses in patients with acute asthma than controls, others studies found a similar detection rate of viruses between children with and without exacerbation [26–29].

The high detection rate of viruses among patients without asthma exacerbation may reflect the increased sensitivity of PCR, which detects nucleic acid from current or previous infections, especially in the case of rhinovirus, which may be present up to 5 to 6 weeks after the beginning of symptomatic infection [30]. Regarding obtaining the sample, previous studies have shown that diagnostic yields of nasopharyngeal swab are comparable to the results obtained with nasopharyngeal aspirate specimens for all of the viruses identified by PCR methods [31–33]. In the present study the rates of virus detection was as high as in previous studies and rhinovirus was the most prevalente, in both group, independent of the sample colletion method.

Although it has been shown that viruses are associated with asthma exacerbations, several studies suggest that other factors, like allergen exposure, in combination with viral infection may increase the risk. In the subjects of this study, according to the results of bivariate analysis some variables were associated with asthma exacerbation: at least one hospitalization for asthma in the past 12 months, at least three emergency room visits in the past 12 months, white ethnicity, monthly family income below 200 US$, cough or dyspnea on exertion, nocturnal cough, exposure to mold and poor medication adherence. After multivariate analysis, the variables that remained associated with a higher risk of exacerbation were at least three emergency visits due to exacerbation in the last 12 months and nonadherence to asthma treatment.

It is known that exposure to allergens in combination with viral infection may increase the likelihood of an exacerbation, and in this population, allergic exposure was more prevalent in the exacerbated group, although without statistical significance. Exposure to mold was associated with exacerbation in the univariate analysis, but this association was not maintained in a multivariate analysis. We know that asthma prevalence in tropical areas is high and it may be linked to exposure to dust mites, parasitic infestation and other unknown aspects [34]. Exposure to molds is associated with allergic diseases, however, it prevalence vary widely probably because of environmental differences, and wide range of diagnostic tests used [2]. We assessed aeroallergen exposure through a questionnaire, not with objective measures, however, the results was similar to data obtained by Brazilian studies, with sensitization prevalence between 46.8% [35] and 54% [36] to at least one aeroallergen in asthmatic children evaluated by skin prick test or standard allergen extracts panel.

In the present study, nonadherence to inhaled corticosteroid was an important risk factor for asthma exacerbation, which indicates that the likelihood of viruses triggering an exacerbation of asthma can be minimized by daily ICS controller therapy. Our results are in accordance with a study in the United Kingdom, in which the regular use of daily ICS controller therapy protected from virus-related asthma exacerbations and hospital admissions [37].

Experimental studies have demonstrated that ICS inhibit respiratory tract viral replication and reduce cytokines in bronchial epithelial cells [38]. These effects of reducing virus-induced inflammation may help to explain the findings observed among patients using ICS in the present study who, despite presence of viral genetic material, showed no clinical evidence of viral disease. This association may have previously gone unnoticed because most of the studies in this area have been performed in countries in which most if not all children with asthma in the studies were on ICS maintenance treatment [7, 10, 14]. In contrast, the present study was conducted in a different setting with a lower rate of ICS use.

This is one of a few studies on viruses related to asthma exacerbations in a low-middle income country and using a control comparison group, which enhances the relevance of the results. This study not only downplays the importance of viruses as the sole culprit in asthma exacerbations, but also highlights the need of a comparison group in similar investigations. Moreover, it is possible that different populations can behave differently in terms of triggering exacerbations and genetic mechanisms may be involved in the association [8, 34]. Thus, larger studies are necessary to provide more insights into the pathogenesis of viral respiratory infections and virus-induced exacerbations of asthma in different populations.

In conclusion, while there is no doubt that viruses are associated with asthma exacerbations, the results of the present study suggest that viral infections per se are insufficient to cause an exacerbation. The proportion of acute respiratory viral infections among children with current asthma exacerbation was similar to that observed among children without exacerbation. The only significant risk factors for acute asthma exacerbations were previous emergency room visits for uncontrolled asthma and nonadherence in asthma treatment. The present study suggests that the occurrence of virus-induced exacerbations in children can be attenuated by the proper use of inhaled corticosteroids.

## Abbreviations

95% CI: 95% confidence interval
ATS/ERS: American Thoracic Society/ European Thoracic Society
FLUVA: Influenza virus A
FLUVB: Influenza virus B
hADV: Human adenovirus
hPIV: Human parainfluenza viruses
hRSV: Human respiratory syncytial virus
ICS: Inhaled corticosteroids
IRR: Incidence risk ratio
PCR: Polymerase chain reaction
RT-PCR: Reverse transcription*-*polymerase chain reaction
